# Supplementary material for: Environmental and genetic regulation of Streptococcus pneumoniae galactose catabolic pathways
Source: Nat Commun. 2024 Jun 17;15:5171. doi: 10.1038/s41467-024-49619-w (PMC11183247; doi:10.1038/s41467-024-49619-w)
Supplement: Supplementary file 1 — Supplementary Information [file 41467_2024_49619_MOESM1_ESM.pdf]

Supplementary Table 1. Oligonucleotide primers used in this study. Bold typeface shows incorporated restriction sites.

**Primers**	**Sequence**
**Real time PCR primers**	
RT_gyrB_F<br>RT_gyrB_R	GGGGCTAGGTGAAATGGACG<br>ACTCACGACGAGGCTCTACT
RT_rgg144_F<br>RT_rgg144_R<br>(SPD0144)	GCTTGTGAGGGTCTAACAGCTTCT<br>CTTGATAGTTGTTGAGCTTGTGCCCA
RT_rgg1518_F<br>RT_rgg1518_R<br>(SPD1518)	TGAACACGGTAAAGTTATTCGCAG<br>CTAGGATGGTATTCGTTGCATAT
RT_rgg0939_F<br>RT_rgg0939_R<br>(SPD0939)	CTGTAGCTGATGAGCATCTTTCA<br>CAGTTTTGGTATAGTCTTCATCGTGT
RT_galK_F<br>RT_galK_R<br>(SPD1634)	ATGAACGTCGTGCTGAGTGT<br>ATGGCGAGCACGTTTCAAAC
RT_lacA_F<br>RT_lacA_R<br>(SPD1053)	GTGCAGATGCTGCAGG<br>GGCCACGAGTCATATAAGC
RT_cps2A_F<br>RT_cps2A_R<br>(SPD0315)	TAACTGCGTTAGTCCTACTAGTTGC<br>ACTGCAAAGAGCGACACAGAGC
RT_pflA_F<br>RT_pflA_R<br>(SPD1774)	GGAAAACCTGTCTGGATTCG<br>TTCACGCCACTTGAACTCAC

RT_pflB_F<br>RT_pflB_R<br>(SPD0420)	GGTATGGGTAACGACGGTCG<br>GTGCATACAGTAGCGACGGA
RT_SPD0066_F<br>RT_SPD0066_R	CACGCATTATGGTTGTAGACGACG<br>ATTTCACACCTGGTGGTGTCGC
RT_SPD0088_F<br>RT_SPD0088_R	GTATTTGGGAACGTGGTTGCCTT<br>CTGAAATTATCGAGTCCGACCCA
RT_SPD0089_F<br>RT_SPD0089_R	TTCGGGTTTGATTGCCCTCTCTTG<br>CCAAATTGGGATGGGAAGAAGCTA
RT_SPD0090_F<br>RT_SPD0090_R	CTGATTCAGGTGACAAACCTGTTAT<br>AGGTATTGGATATCCAATTTGGCAC
RT_CodY_F<br>RT_CodY_R<br>(SPD1412)	CGCGTCAGTTAGCGGATATTATTCATT<br>GAAGAATTGCTCTACGCGATCTGTA
RT_SPD1039_F<br>RT_SPD1039_R	CAGCCGGATTTGTCATTTGAGACTAT<br>GTACCTACTGCTTTCTCGCGAATAA
Construction of LacZ reporter strains	
P*lacA*-Fiusion-F	GAC**GCATGC**GCCAATCAGGTTGAAGAATTAG
P*lacA*-Fiusion-R	ACG**GGATCC**GCATCTGCACCGATAACAATAG
P*galK*-Fiusion-F	GAC**GCATGC**TGTCTGGTTTCTTCTGTAACAG

P*galK*-Fiusion-R	ACG**GGATCC**CAGCAGTAAGATGTTGTGTC
Construction of Luc reporter strains	
PlacA Fusion-F	GAC**GCATGC**GCCAATCAGGTTGAAGAATTAG
PlacAR Fusion-R	ACG**GGATCC**GCATCTGCACCGATAACAATAG
PgalK Fusion-F	GAC**GCATGC**TGTCTGGTTTCTTCTGTAACAG
PgalK Fusion-R	ACG**GGATCC**CAGCAGTAAGATGTTGTGTC

Supplementary Figure 1:

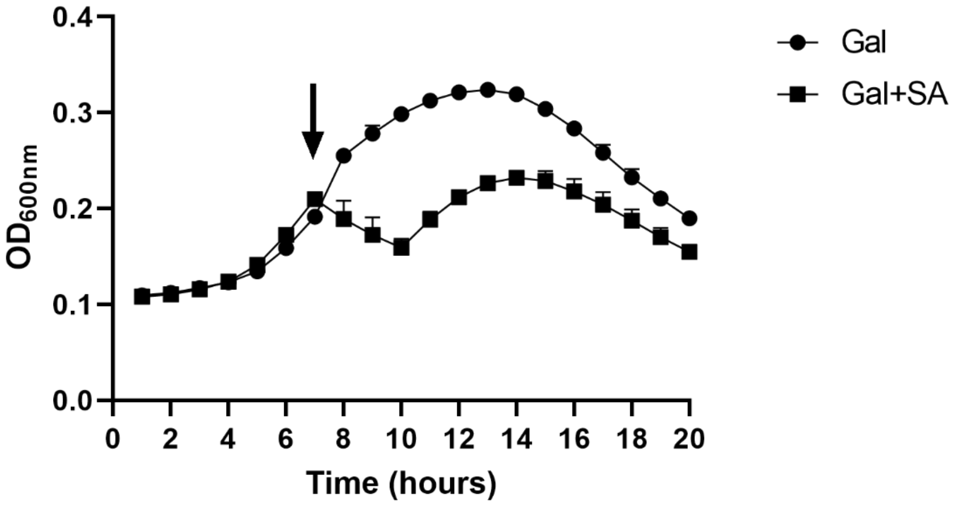

Supplementary Figure 1: The addition of 3.5 mM ManNAc represses the pneumococcal growth on galactose (Gal). Downward arrow shows when sialic acid (SA) was added. Mean of four independent experiments, each in duplicate, with their SEM is given.

Supplementary Figure 2

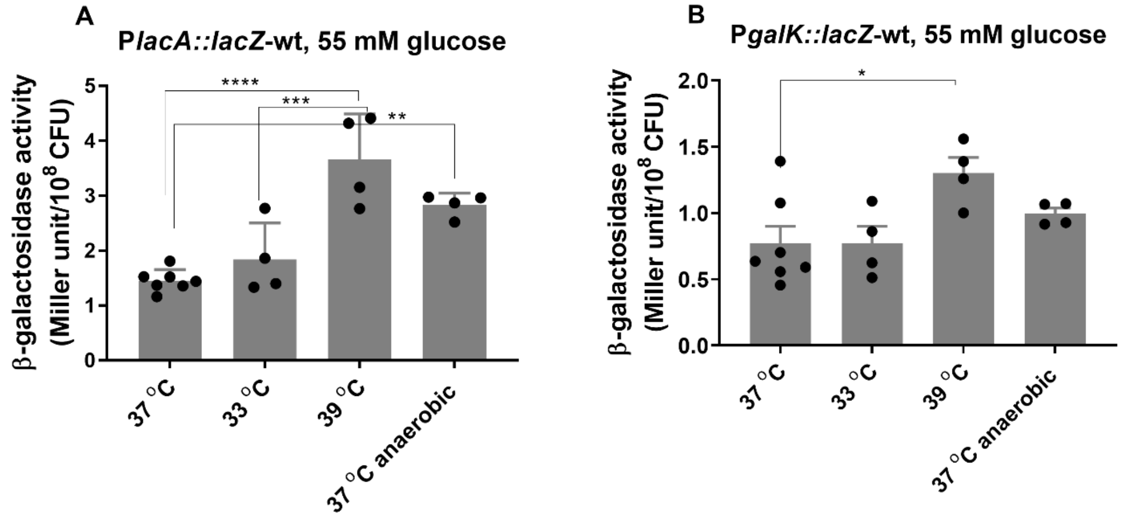

Supplementary Figure 2: Impact of environmental factors on the expression of galactose catabolic pathways in the presence of glucose. Analysis of P*lacA* (A) and P*galK* (B) induction in CDM supplemented with 55mM glucose incubated 4 hours microaerobically at different temperature or anaerobically at 37°C using LacZ reporter assay. Mean of at least four independent experiments with their SEM is given. Statistical analysis was performed using One-way ANOVA followed by Tukey's multiple comparisons test, *p=0.028, **p=0.0026, ***p=0.0006, ****p<0.0001.
