# Supplementary material for: Environmental and genetic regulation of Streptococcus pneumoniae galactose catabolic pathways
Source: Nat Commun. 2024 Jun 17;15:5171. doi: 10.1038/s41467-024-49619-w (PMC11183247; doi:10.1038/s41467-024-49619-w)
Supplement: Supplementary file 3 — Reporting Summary [file 41467_2024_49619_MOESM3_ESM.pdf]

nature portfolio

Corresponding author(s): Hasan Yesilkaya

Last updated by author(s): May 29, 2024

Reporting Summary

Nature Portfolio wishes to improve the reproducibility of the work that we publish. This form provides structure for consistency and transparency in reporting. For further information on Nature Portfolio policies, see our Editorial Policies and the Editorial Policy Checklist.

Statistics

For all statistical analyses, confirm that the following items are present in the figure legend, table legend, main text, or Methods section.

n/a	Confirmed	
☐	☒	The exact sample size (*n*) for each experimental group/condition, given as a discrete number and unit of measurement
☐	☒	A statement on whether measurements were taken from distinct samples or whether the same sample was measured repeatedly
☐	☒	The statistical test(s) used AND whether they are one- or two-sided<br>*Only common tests should be described solely by name; describe more complex techniques in the Methods section.*
☒	☐	A description of all covariates tested
☒	☐	A description of any assumptions or corrections, such as tests of normality and adjustment for multiple comparisons
☐	☒	A full description of the statistical parameters including central tendency (e.g. means) or other basic estimates (e.g. regression coefficient) AND variation (e.g. standard deviation) or associated estimates of uncertainty (e.g. confidence intervals)
☐	☒	For null hypothesis testing, the test statistic (e.g. *F*, *t*, *r*) with confidence intervals, effect sizes, degrees of freedom and *P* value noted<br>*Give P values as exact values whenever suitable.*
☒	☐	For Bayesian analysis, information on the choice of priors and Markov chain Monte Carlo settings
☒	☐	For hierarchical and complex designs, identification of the appropriate level for tests and full reporting of outcomes
☒	☐	Estimates of effect sizes (e.g. Cohen's *d*, Pearson's *r*), indicating how they were calculated

Our web collection on statistics for biologists contains articles on many of the points above.

Software and code

Policy information about availability of computer code

Data collection	Not applicable
Data analysis	Data analysis was done using GraphPad Prism version 9.4 (Dotmatics, Boston, MA, USA).

For manuscripts utilizing custom algorithms or software that are central to the research but not yet described in published literature, software must be made available to editors and reviewers. We strongly encourage code deposition in a community repository (e.g. GitHub). See the Nature Portfolio guidelines for submitting code & software for further information.

Data

Policy information about availability of data

All manuscripts must include a data availability statement. This statement should provide the following information, where applicable:

- Accession codes, unique identifiers, or web links for publicly available datasets
- A description of any restrictions on data availability
- For clinical datasets or third party data, please ensure that the statement adheres to our policy

All data supporting the paper’s conclusions are included in the main text and supplementary material file. The RNA-seq data is available from the Gene Expression Omnibus (GEO) repository with the primary accession code GSE246424.

Research involving human participants, their data, or biological material

Policy information about studies with human participants or human data. See also policy information about sex, gender (identity/presentation), and sexual orientation and race, ethnicity and racism.

Reporting on sex and gender	Not applicable
Reporting on race, ethnicity, or other socially relevant groupings	Not applicable
Population characteristics	Not applicable
Recruitment	Not applicable
Ethics oversight	Not applicable

Note that full information on the approval of the study protocol must also be provided in the manuscript.

Field-specific reporting

Please select the one below that is the best fit for your research. If you are not sure, read the appropriate sections before making your selection.

☒ Life sciences ☐ Behavioural & social sciences ☐ Ecological, evolutionary & environmental sciences

For a reference copy of the document with all sections, see nature.com/documents/nr-reporting-summary-flat.pdf

Life sciences study design

All studies must disclose on these points even when the disclosure is negative.

Sample size	The number of animals per cohort is based on our long-term experience of working with mouse models of pneumococcal disease and colonization and statistical analysis of the data. This experience and analyses show that group sizes of between 10 and 20 animals are required to detect statistically significant differences in the pneumonia model and 5 and 10 animals at each time point are needed to detect statistically significant differences in colonization model. The power of the proposed study has been calculated (http://biomath.info/power/ttest.htm). Based on published data, we have calculated the following sample sizes. For a study with 90% power at significance level of 0.05, 10 animals detect a difference of 30 hours in survival with a standard deviation of 20 hours (assuming equal variance in the two groups).
Data exclusions	All data has been included except in cases where the model deemed not established due to the technical issues such as operator error.
Replication	All attempts for replication was successful.
Randomization	We used Excel software to generate a column of random numbers which were use to randomly allocate animals to each group.
Blinding	The investigators were blinded to group allocation and data analysis.

Reporting for specific materials, systems and methods

We require information from authors about some types of materials, experimental systems and methods used in many studies. Here, indicate whether each material, system or method listed is relevant to your study. If you are not sure if a list item applies to your research, read the appropriate section before selecting a response.

Materials & experimental systems

n/a	Involved in the study	
☒	☐	Antibodies
☒	☐	Eukaryotic cell lines
☒	☐	Palaeontology and archaeology
☐	☒	Animals and other organisms
☒	☐	Clinical data
☒	☐	Dual use research of concern
☒	☐	Plants

Methods

n/a	Involved in the study	
☒	☐	ChIP-seq
☒	☐	Flow cytometry
☒	☐	MRI-based neuroimaging

Animals and other research organisms

Policy information about studies involving animals; ARRIVE guidelines recommended for reporting animal research, and Sex and Gender in Research

Laboratory animals	Mouse, CD1 or MF1 , 8-10 weeks
Wild animals	The study did not involve wild animals
Reporting on sex	The results apply to female mice. Sex was not considered as variable. We used female mice because we optimized our model using female mice.
Field-collected samples	We did not use field collected samples.
Ethics oversight	Mouse studies were performed under the project (permit no. PP0757060) and personal (permit no. 80/10279) licenses according to the United Kingdom Home Office guidelines under the Animals Scientific Procedures Act 1986, and the University of Leicester ethics committee approval. The protocol used was approved by both the U.K. Home Office and the University of Leicester ethics committee

Note that full information on the approval of the study protocol must also be provided in the manuscript.

Plants

Seed stocks	Not applicable
Novel plant genotypes	Not applicable
Authentication	Not applicable
